# Skeletal Status, Body Composition, and Glycaemic Control in Adolescents with Type 1 Diabetes Mellitus

**DOI:** 10.1155/2018/8121634

**Published:** 2018-09-03

**Authors:** Elzbieta Wierzbicka, Anna Swiercz, Pawel Pludowski, Maciej Jaworski, Mieczyslaw Szalecki

**Affiliations:** ^1^Department of Human Nutrition, Warsaw University of Life Sciences (SGGW), Warsaw, Poland; ^2^Department of Endocrinology and Diabetology, The Children's Memorial Health Institute, Warsaw, Poland; ^3^Department of Biochemistry, Radioimmunology, and Experimental Medicine, The Children's Memorial Health Institute, Warsaw, Poland; ^4^Faculty of Medicine and Health Sciences, Jan Kochanowski University, Kielce, Poland

## Abstract

**Background:**

Disturbed bone turnover, osteoporosis, and increased fracture risk are late complications of insulin-dependent diabetes mellitus. Little is known about how far and to what extent can glycaemic control of type 1 diabetes mellitus (T1DM) prevent disturbances of bone health and body composition during the growth and maturation period.

**Objective:**

The aim of this cross-sectional study was to compare the skeletal status outcomes and body composition between patients stratified by glycaemic control (1-year HbA1c levels) into well- and poorly-controlled subgroups in a population of T1DM adolescents, that is, <8% and ≥8%, respectively.

**Subjects and Methods:**

Skeletal status and body composition were evaluated in 60 adolescents with T1DM (53.3% female; mean aged: 15.1 ± 1.9 years; disease duration: 5.1 ± 3.9 years) using dual energy X-ray absorptiometry (GE Prodigy). The results were compared to age- and sex-adjusted reference values for healthy controls. The calculated Z-scores of different metabolic control subgroups were compared. Clinical data was also assessed.

**Results:**

As evidenced by Z-scores, patients with T1DM revealed a significantly lower TBBMD (total body bone mineral density), TBBMC (total body bone mineral content), S24BMD (bone mineral density of lumbar spine L2–L4), and TBBMC/LBM ratio (total body bone mineral content/lean body mass), but higher FM (fat mass) and FM/LBM ratio (fat mass/lean body mass) values compared to an age- and sex-adjusted general population. The subset (43.3% patients) with poor metabolic control (HbA1c ≥ 8%) had lower TBBMD, TBBMC, and LBM compared to respective values noted in the HbA1c < 8% group, after adjusting for confounders (mean Z-scores: −0.74 vs. −0.10, *p* = 0.037; −0.67 vs. +0.01, *p* = 0.026; and −0.45 vs. +0.20, *p* = 0.043, respectively). Additionally, we found a significant difference in the TBBMC/LBM ratio (relative bone strength index) between the metabolic groups (−0.58 vs. −0.07; *p* = 0.021). A statistically significant negative correlation between 1-year HbA1c levels and Z-scores of TBBMD, TBBMC, and LBM was also observed. In patients with longer disease duration, a significant negative correlation was established only for TBBMD, after adjusting for confounders. The relationships between densitometric values and age at onset of T1DM and sex were not significant and showed no relation to any of the analysed parameters of the disease course.

**Conclusion:**

Findings from this study of adolescents with T1DM indicate that the lower Z-scores of TBBMD, TBBMC, and LBM as well as the TBBMC/LBM ratio are associated with increased HbA1c levels. Their recognition can be crucial in directing strategies to optimise metabolic control and improve diabetes management for bone development and maintenance in adolescents with T1DM.

## 1. Introduction

Type 1 diabetes mellitus (T1DM) is one of the most common chronic childhood diseases, the prevalence of which is rising globally [1, 2]. In Poland, a recent epidemiological study confirmed this trend and demonstrated an increased incidence of diabetes in the paediatric population [3, 4]. Type 1 DM is a chronic systemic autoimmune-mediated disease characterised by hyperglycaemia, due to progressive absolute insulin deficiency, as a result of the destruction of the pancreatic *β*-cells [1, 5]. In the majority of patients, the disease develops during childhood and patients are therefore exposed to the deleterious effects of insulin deficiency and hyperglycaemia for a long time, causing micro- and macrovascular complications [6, 7]. It is apparent that the skeleton has been recognised as an organ that is also adversely affected by a diabetic condition [8–12]. The reported results indicate that diabetic bone disease with fragility is another complication of a long-standing disease in patients with T1DM [13, 14]. There is evidence confirming an association between glucose utilisation and bone-fat tissue interaction [15–18], as well as the muscle-bone crosstalk [19–21]. The risk of fracture among adult patients with long-standing T1DM is higher compared with an age- and sex-matched non-diabetic control population [22–24], while no relevant data exist on childhood type 1 DM. Notably, one study reported, for the first time, a higher fracture incidence in young males and females with T1DM (aged < 20 years) compared to subjects without T1DM, as assessed by hazard ratio (HR) — 1.14 (95% CI 1.01–1.29) and 1.35 (95% CI 1.12–1.63), respectively [25]. On the other hand, recent studies have reported low rates of non-vertebral fractures in aging individuals with long-standing T1DM [26]. However, this study described an association between the presence of cardiovascular disease and low bone mineral density at the femoral neck. Other studies, looking specifically at adolescents [27] and young women [28] as well as adult T1DM patients [29, 30], identified microvascular complications as a factor of skeletal deficits and decreased bone quality.

Childhood and adolescence are the most critical periods for growth and bone mineral accrual [31–33]. Importantly, the onset of the disease often starts at a time when peak bone mass is not yet achieved; thus, it is likely that it impairs bone accrual during this period of bone growth [34]. In children with T1DM, abnormal bone accrual (density and quality) [10–12] can also be associated with alterations of the growth hormone/insulin like growth factor-1 (GH/IGF-1) axis [35]. Since the maximum bone mineral accrual occurs during the first two decades of life [31, 32], the reduced bone mineral mass gain during these critical periods can lead to serious consequences, in particular, osteoporosis and bone fracture risk in later in life [33]. In type 1 DM, a failure of bone cells in high-glucose conditions [36] is associated with abnormal activities of neighbouring cells in bone, such as adipocytes, mesenchymal cells, and endothelial progenitor cells [37], and may increase influence on adipocyte differentiation and fat accumulation [16–18], as well as increased bone marrow adiposity [15, 38]. Adipose tissue is a metabolically dynamic organ [39] that is also an endocrine organ, capable of producing cytokines and adipokines that may regulate bone metabolic homeostasis [10, 13, 14]. Due to the fact that adipose tissue distribution affects bone mass, the relationship between adipose and bone tissue is notable [15, 16, 18]. On the other hand, deteriorated muscle function [40] might result in adverse effects on skeletal status [19–21]. Poor glycaemic control, as seen in hyperglycaemia, is a known risk factor for bone disorders [36, 37]. Many substantial studies in patients with T1DM described an association with reduced bone mineral density and osteoporosis [8, 9]. Bone mass and density deficits in T1DM patients most likely reflect the defects in bone formation with osteoblastic dysfunction [10, 11], as alterations of bone microarchitectures are involved in reducing bone quality and strength [12–14]. This, in turn, increases the risk of fractures in later life [22–25]. It is important to note that in children and adolescents with T1DM such data is limited and inconsistent [34].

In this context, the hypothesis of this study is that inadequate glycaemic control is an important variable associated with insufficient bone mineral accrual, and that this association is related to decreased muscle mass and increased fat stores, which may be an important factor in bone development in childhood type 1 DM.

The aim of this cross-sectional study was to compare the skeletal status outcomes, body composition, and relative bone strength between patients stratified by glycated haemoglobin levels (%HbA1c) into two study subgroups, that is, relatively well- and poorly-controlled in a population based on T1DM adolescents.

## 2. Material and Methods

### 2.1. Study Population

The purposive cross-sectional study population was composed of 60 Caucasian adolescent patients (aged 15.1 ± 1.9 years) with type 1 insulin-dependent diabetes mellitus (T1DM), admitted for a regular check-up to the Department of Endocrinology and Diabetology of the Children's Memorial Health Institute in Warsaw, Poland. Participants were selected based on the following criteria: age of 12–18 years, males and females (1 : 1 ratio), diagnosis of T1DM according to ISPAD criteria, duration of diabetes and received medical services in the clinic for at least six months, treated by continuous subcutaneous insulin infusion, complete data for the glycated haemoglobin (A1c) values, and the DXA measurements performed. The exclusion criteria for patients were as follows: history of any acute (severe hypoglycaemia, diabetic ketoacidosis) or chronic (retinopathy, neuropathy, and nephropathy) complications of diabetes, the presence of any associated metabolic bone or musculoskeletal diseases, and any chronic illness other than diabetes as well as any medications other than insulin.

The study was approved by the local ethics committee (Warsaw, Poland) and was conducted in accordance with the World Medical Association Declaration of Helsinki. Written informed consent was obtained from the parents of all the patients at the initial enrolment in this study.

### 2.2. Data Collection

For all study participants, a medical record review was undertaken. The following information was collected and recorded using a standardised questionnaire: demographics, physical activity (including school-based physical activity), age at diabetes onset, duration of diabetes, insulin delivery system, daily insulin dose (units per kilogram of body weight), general medical history, and clinical characteristics, as well as data related to metabolic control of diabetes, including the glycated haemoglobin (HbA1c) level in a 1-year period.

Each patient underwent a comprehensive examination that included a measurement of skeletal and body composition using dual-energy X-ray absorptiometry (DXA), allowing the assessment of three tissue compartments (bone, lean, and fat tissue). Anthropometric measurements of the patients were obtained together with densitometry during a single visit. The adolescents' height and weight Z-scores were calculated using national reference data. The subjects' pubertal stage was assessed by a physical examination performed by a trained paediatrician using the Tanner classification.

Blood samples were obtained in the morning after an overnight fast. Glycated haemoglobin A1c (HbA1c) was analysed in a whole blood sample by a direct turbidimetric inhibition immunoassay (Roche Diagnostics, Germany) that determines HbA1c as a percentage of total haemoglobin (HbA1c, %), performed according to IFCC guidelines. The serum levels of calcium and inorganic phosphorous were measured spectrophotometrically using commercially available test kits (Roche Diagnostics, Germany). Serum total 25(OH)D and PTH (iPTH) were measured by a chemiluminescence immunoassay (ECLIA, Roche Diagnostics, Germany), according to the manufacturer's instructions.

### 2.3. Assessment of Glycaemic Control

Glycaemic control was evaluated on the basis of %HbA1c levels in the past year. These were calculated as an average of data collected at least three times in a 1-year period. Based on HbA1c levels, T1DM patients with different metabolic control states were divided into subgroups. For this purpose, patients with HbA1c < 8% (<64 mmol/mol) were considered as having relatively good glycaemic control, while those with HbA1c ≥ 8% (≥64 mmol/mol) as having poor glycaemic control.

### 2.4. Assessment of Skeletal Status and Body Composition — DXA Data

In all investigated patients, skeletal data, that is, total body bone mineral density (TBBMD, g/cm^2^), total body bone mineral content (TBBMC, g), and both bone mineral density (S24BMD, g/cm^2^) and bone mineral content (S24BMC, g) at the lumbar spine L2–L4, as well as body composition (lean mass and fat mass of the total body) were measured by dual-energy X-ray absorptiometry (DXA), using the Prodigy Advance device (General Electric™) with paediatric software ver. 14. For the calibration of a densitometer, a daily quality control procedure was performed. Additionally, anthropomorphic spine phantom was scanned at least twice a week. Measurement error (CV%) was 0.33% for L2–L4 BMD and 0.35% for L2–L4 BMC in the whole study period.

Moreover, the fat mass/lean body mass ratio (FM/LBM ratio) was calculated from DXA data. Also, relative bone strength index, estimated as the total body bone mineral content/lean body mass ratio (TBBMC/LBM ratio), and bone mineral content of lumbar spine L2–L4/lean body mass (S24BMC/LBM ratio) were considered as the muscle-bone relationship indicator.

Absolute densitometric data were compared with sex- and age-adjusted reference values established in healthy counterparts [41, 42], and the Z-scores were calculated using the following formula: Z‐score = [(result for a subject)–(age‐ and gender‐matched mean in reference subjects)]/(age‐ and gender‐matched standard deviation in reference subjects). Low bone mass or bone mineral density was defined as Z-scores lower than or equal to −2.0 standard deviation [43].

### 2.5. Statistical Analysis

For the purposes of this study, multiple analyses were performed. In the first one, the results were referred to the norms according to age and sex and presented as Z-score (corresponding abbreviations are preceded by the letter “Z”). Deviations from the normal distribution were verified by the Shapiro-Wilk test. Z-score values were evaluated by one-sample Student's *t*-test with the hypothetic value “0”, assumed to reflect expected data in healthy control subjects. Only for fat mass (FM) was the non-parametric one-sample Wilcoxon test used. Association tests (Student's *t*-test or Mann–Whitney's *U* test) were performed to compare the quantitative variables in both subgroups. The differences in descriptive values were estimated using the Chi-square test for categorical variables. Pearson's correlation coefficient or nonlinear association Spearman's correlation coefficient were calculated to assess the relationships between skeletal and body composition parameters and clinical data. Correlation analysis was also performed between disease duration, insulin requirement, glycemic control (%HbA1c levels), and skeletal and body composition Z-score values (for age- and sex-dependent reference data for healthy subjects). Partial correlation coefficients were calculated for these parameters, using age, sex, pubertal stage, height, weight, and physical activity level as confounders. The second analysis consisted of comparison of Z-score values for bone density and body composition parameters, between the two subgroups of different metabolic control, that is, HbA1c < 8% and ≥8%. Analysis of covariance (ANCOVA) was used to compare skeletal and body parameters between subgroups of good and poor metabolic control, adjusting for disease duration, age, sex, pubertal stage, insulin dose, height, weight, and physical activity level. The analyses were performed using software Statistica v.13 and IBM SPSS Statistics v.25. Data were presented as mean ± SD (standard deviation) and SE (standard error of the mean) or number (%) unless specified, and a *p*-value ≤ 0.05 was considered to indicate statistical significance.

## 3. Results

### 3.1. Characteristics of the Studied Group

The study included 60 adolescents with type 1 diabetes (53.3% female) (mean ± SD) aged 15.0 ± 1.9 years with a disease duration of 5.1 ± 3.9 years and patients with young-onset diabetes of 9.9 ± 3.9 years. Of all DM patients, 56.7% of the patients were categorised as relativity well-controlled (HbA1c < 8%; 64 mmol/mol) and 43.3% of the patients as poorly-controlled (HbA1c ≥ 8%; 64 mmol/mol). The basic characteristics of both DM subgroups are given in Table 1.

Overall, the mean HbA1c value (in a 1-year period) was 7.9 ± 1.4% (Table 1). The highest HbA1c levels were observed in the uncontrolled DM subgroup (9.2 ± 1.0%), while the well-controlled DM subgroup showed the lowest HbA1c levels (6.9 ± 0.6%); *p* = 0.001. Patients from both subgroups had a similar age at the diagnosis of about 10 years; there were no statistically significant differences. The group of adolescents with poor glycaemic control was characterised by significantly longer diabetes duration (6.7 ± 4.3 vs. 3.9 ± 3.1 years, *p* = 0.005), higher insulin dose (0.86 ± 0.22 vs. 0.67 ± 0.16 U/kg bw/d, *p* = 0.005) which is strictly connected with HbA1c levels, older age (16.0 ± 1.7 vs. 14.4 ± 1.8 years, *p* = 0.001), and lower body height values (SD score) (i.e., −0.34 ± 1.02 vs. +0.38 ± 1.10, *p* = 0.012), compared to patients with good glycaemic control. Both studied groups revealed low 25(OH)D serum levels of 15.3 ± 7.0 ng/mL, below the optimal values of 30–50 ng/mL (Table 1). Significantly increased serum levels of 25(OH)D levels (17.2 ± 7.9 ng/mL vs. 12.8 ± 4.4 ng/mL; *p* = 0.015) were found in the well-controlled patients compared to those found in the uncontrolled DM subgroup. The serum calcium, iPTH, and inorganic phosphorus levels were within the normal range in both groups (*p* > 0.05).

Among the studied subgroups (Table 1), no significant differences were found in the remaining analysed clinical and other anthropometric data. Similarly, no differences were found in DXA parameters (Table 1), expressed as absolute values. In contrast, the analysis of the skeletal status and body composition in comparison with Z-scores, calculated according to age- and gender-dependent reference data for the healthy subject, revealed statistically significant differences between the examined groups of subjects (Table 2).

### 3.2. Skeletal Status and Body Composition

Skeletal status and body composition of T1DM patients, as a whole group, as well as in the well-controlled subgroup (HbA1c < 8%) and the poorly-controlled subgroup (HbA1c ≥ 8%), were referenced to their age- and gender-matched healthy counterparts, as shown in Table 2. As indicated in Table 2, T1DM patients as a whole had slightly decreased Z-scores for bone mass and density of the whole skeleton (TBBMD, TBBMC) and slightly decreased bone density in the lumbar spine (S24BMD) region, compared to the value of zero (expected in healthy counterparts). In contrast, the HbA1c ≥ 8% subgroup (Table 2) revealed decreased Z-scores for bone density of both whole skeleton and lumbar spine (TBBMD Z-score of −0.74 ± 0.80, *p* < 0.01 and S24BMD Z-score of −0.43 ± 0.91, *p* < 0.05). Bone mineral content appeared decreased in the whole skeleton (TBBMC Z-score) but normal in the lumbar spine (S24BMC Z-score).

**Figure 1:**
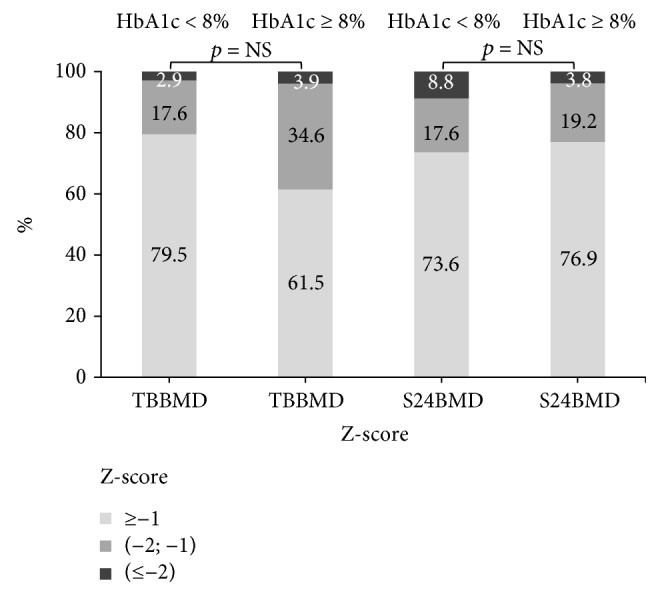
The stacked bar of frequency distribution (%) of the bone mineral density of the whole skeleton and of the lumbar spine bone mineral density in T1DM patients with well- or poorly controlled glycaemia. TBBMD — total body bone mineral density; S24BMD — bone mineral density, lumbar spine L2–L4.

Figure 1 shows BMD Z-score distribution in relation to glycaemic control. As shown in Figure 1, the majority of cases, irrespective of %HbA1c value or region of the skeleton, had a BMD Z-score above the value of −2.00.

Lean body mass (an estimate of muscle mass) Z-score values (Table 2) calculated for the whole group appeared normal (mean LBM Z-score of −0.08 ± 0.90), but FM and the distributed relationship between FM and LBM as assessed by the FM/LBM ratio were slightly higher (FM Z-scores of +0.38 ± 1.27, *p* < 0.05; FM/LBM Z-scores of +0.31 ± 0.99; *p* < 0.05) when compared to the value of zero (expected in healthy counterparts). The HbA1c ≥ 8% subgroup had decreased LBM Z-score values of −0.45 ± 0.77 (*p* < 0.05), normal FM (FM Z-score of +0.40 ± 1.03, *p* = 0.060), and statistically increased FM/LBM Z-score values of +0.45 ± 0.90 (*p* < 0.05) (Table 2).

Relative bone strength was estimated as the total body bone mineral content/lean body mass ratio. Patients with T1DM had TBBMC/LBM ratio Z-scores slightly lower than expected in healthy subjects, but S24BMC/LBM ratio Z-scores were normal compared to their age- and gender-matched healthy reference (Table 2). Furthermore, when HbA1c (%) was controlled for, the subgroup with HbA1c < 8% showed physiological values for all DXA measured parameters, and S24BMC/LBM Z-score was even slightly higher than expected in the healthy subjects. In contrast, the HbA1c ≥ 8% subgroup revealed decreased Z-scores for the whole skeleton (TBBMC Z-score) but normal in the lumbar spine (S24BMC Z-score) and had decreased LBM Z-scores. In these patients, there was a significant relationship between BMC and LBM, where the TBBMC/LBM ratio value was lower than the value of zero, as assessed by the ratio Z-score of −0.58 ± 0.92 (*p* < 0.05), but the S24BMC/LBM ratio Z-score was normal and not significant (Table 2).

### 3.3. Effect of Disease Duration, Insulin Requirement, and HbA1c Levels

We explored the effects of disease-related factors, such as disease duration, insulin requirement, and HbA1c (%), on skeletal and body composition parameters (Z-scores) by carrying out a partial correlation analysis (controlled for age, sex, pubertal stage, height, weight, and physical activity level). As shown in Table 3, we detected no effect of disease duration on the studied DXA parameters, except for TBBMD Z-score value (*p* < 0.05). Moreover, TBBMC and LBM Z-scores tended to be lower as the disease duration increased (*p* = 0.056 and *r*, respectively). In contrast, diabetes duration was positively related to insulin requirement and HbA1c levels (*p* < 0.05). As presented in Table 3, the insulin requirement dose was negatively associated with Z-scores for TBBMD, TBBMC, TBBMC/LBM, S24BMD, and 24TBBMC/LBM (*p* < 0.05). Mean 1-year HbA1c levels (%) were also negatively related to the Z-scores for bone mass and density of the whole skeleton (TBBMD, TBBMC) and LBM (*p* < 0.05).

**Figure 2:**
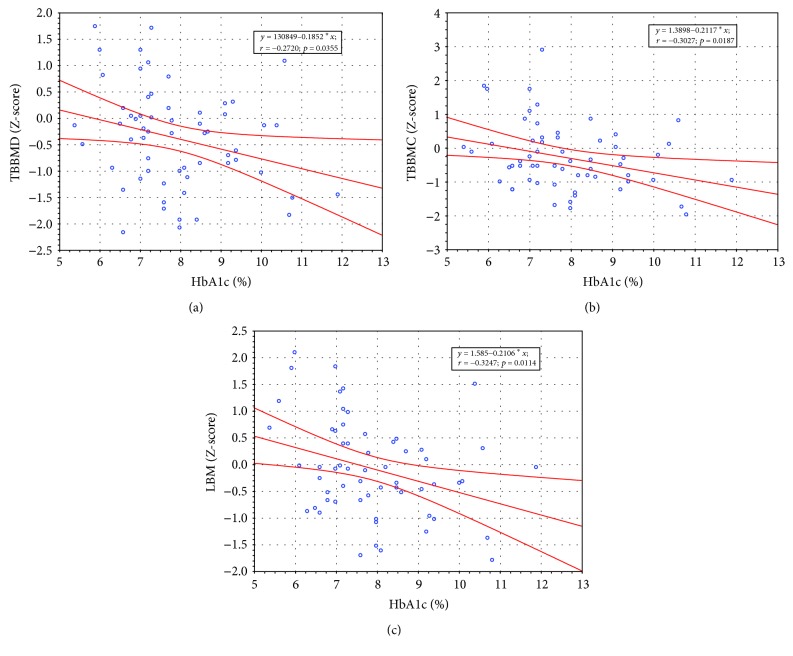
HbA1c (%) levels in relation to Z-score values of (a) the total body bone mineral density, (b) total bone mineral content, and (c) lean body mass in patients with T1DM. TBBMD — total body bone mineral density; TBBMC — total body bone mineral content; LBM — lean body mass.

As shown in Figure 2, HbA1c (%) levels correlated negatively with Z-scores for TBBMD, TBBMC, and LBM (*r* = −0.27, −0.30, and −0.32; *p* < 0.05, respectively) as well as Z-scores for absolute TBBMC (*r* = −0.28, *p* = 0.038) and LBM (*r* = −0.27, *p* = 0.040) (data not shown). No significant relationships were found between HbA1c levels (%) and other DXA parameters as well as Z-scores and absolute data (*p* > 0.05).

### 3.4. Skeletal Status and Body Composition in Relation to Glycaemic Control

**Figure 3:**
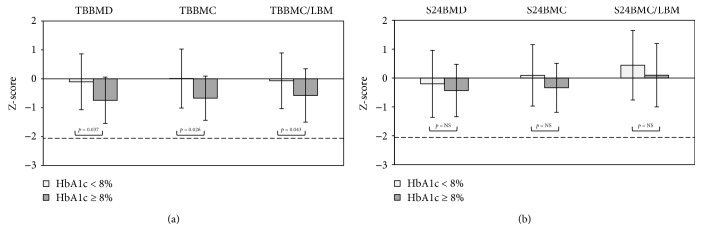
Comparison of DXA parameters expressed as the mean (±SD) Z-score values (calculated according to age- and gender-dependent data for healthy subjects), including (a) bone mineral density and bone mineral content of the whole skeleton, relative bone strength ratio (TBBMC/LBM ratio), and (b) bone mineral density and bone mineral content of the lumbar spine, between T1DM patients with well-controlled glycaemia versus those with poorly controlled glycaemia. The dotted line represents the lowest level of the acceptable range of value in healthy subjects (Z-score = −2.0). *p*-Value indicates significant differences between studied subgroups, adjusted for age, sex, disease duration, pubertal stage, insulin dose, height, weight, and physical activity level; NS, not significant. TBBMD — total body bone mineral density; TBBMC — total body bone mineral content; TBBMC/LBM — total body bone mineral content/lean body mass ratio; S24BMD — bone mineral density, lumbar spine L2–L4; S24BMC — bone mineral content, lumbar spine L2–L4; S24BMC/LBM — bone mineral content, lumbar spine L2–L4/lean body mass ratio.

**Figure 4:**
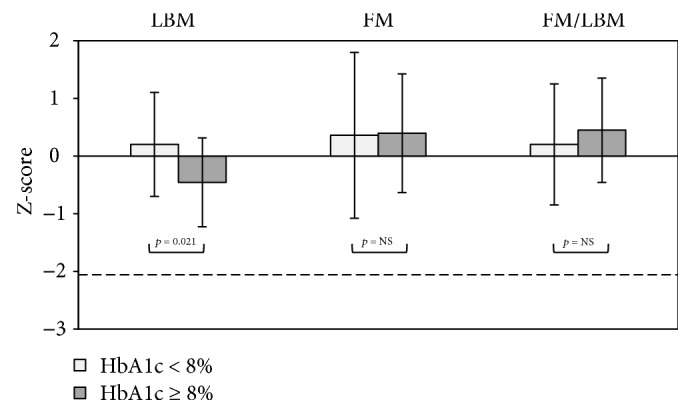
Comparison of body composition parameters expressed as the mean (±SD) Z-score values (calculated according to age- and gender-dependent data for healthy subjects), including lean body mass, fat body mass, and FM/LBM ratio, between T1DM patients with well-controlled glycaemia versus those with poorly controlled glycaemia. The dotted line represents the lowest level of the acceptable range of value in healthy subjects (Z-score = −2.0). *p*-Value indicates significant differences between the studied subgroup adjusted for age, sex, disease duration, pubertal stage, insulin dose, height, weight, and physical activity levels; NS, not significant. LBM — lean body mass; FM — fat mass; FM/LBM — fat mass/lean body mass ratio.

As shown in Figures 3 and 4, skeletal and body composition parameters were compared between the HbA1c < 8% and HbA1c ≥ 8% subgroups. After adjusting for disease duration, age, sex, pubertal stage, insulin dose, height, weight, and physical activity level, significantly lower Z-scores were noted in the HbA1c ≥ 8% subgroup for TBBMD and TBBMC, as well as for the TBBMC/LBM ratio (Figure 3). In the lumbar spine, the Z-score values showed that, in T1DM patients with HbA1c 0> 8%, S24BMD and S24BMC/LBM were slightly lower when compared with the HbA1c < 8% subgroup; however, the differences were not significant (*p* > 0.05). Moreover, S24BMC Z-scores tended to be lower in HbA1c ≥ 8% than in the HbA1c < 8% subgroup (*p* = 0.098). In the comparison of body composition parameters (Figure 4), we found that only Z-scores of LBM in the HbA1c ≥ 8% subgroup were lower than those with good metabolic control (*p* < 0.05). There were no significant relationships found between DXA values and age at T1DM diagnosis and sex (*p* > 0.05).

## 4. Discussion

To the best of our knowledge, this study for the first time provided comprehensive analyses of skeletal status, body composition, and estimates of relative bone strength in adolescents with T1DM using the DXA method as well as the normative reference data of a nationally representative group of healthy adolescents in applying the DXA system. Moreover, our results enabled us to evaluate the possible impact of glycaemic control on the three main compartments of the human body: bone, fat, and muscle mass, as well as the functional relationships between the abovementioned tissues.

Glycated haemoglobin A1c (HbA1c, %) is currently used as a measure of longer-term glycaemic control. This value reflects average blood glucose levels over a period of two to three months, and is an indicator for overall glucose exposure integrating both fasting and postprandial blood glucose levels. Regular HbA1c measurement is recommended by international guidelines for the assessment of glycaemic control as it provides information on long-term glycaemic status [5]. Several studies confirmed a link between elevated HbA1c levels and development of microvascular and macrovascular complications [6, 7]. Therefore, achieving low HbA1c levels is an important therapeutic target in the management of diabetes [5]. To investigate whether metabolic control is associated with skeletal status outcomes, body composition, and relative bone strength in adolescents with T1DM, two subgroups of patients were identified as having good (HbA1c < 8%) or poor glycaemic control (HbA1c ≥ 8%). The concept of stratifying the patients into these two groups is based on the fact that HbA1c levels impact the presence of diabetic complication [7]. The maintenance of glycaemic control levels under the 8% value in this study was utilised in an attempt to examine the effects of glycaemic management on the skeletal status and body composition in patients with T1DM. It was hypothesised that improved glycaemic control would prevent the negative effects of T1DM on bone, muscle, and fat tissues in adolescents with T1DM. While poorer glycaemic control and bone complications have already been described in the literature [8–12], the evidence examining the skeletal status and body composition in adolescents with T1DM is limited [16, 17, 20, 27, 34].

In the present study, it was proven that adolescents with T1DM in the poor metabolic control group had significantly lower BMD and BMC Z-score values for the total skeleton and lower BMD Z-score values for the lumbar spine. Several previous studies in T1DM children and adolescents also investigated this association and found similar results. In particular, multiple cross-sectional studies using DXA-based outcomes reported that bone mineral density and/or bone mineral content were significantly lower in T1DM patients when compared to non-diabetic subjects [44–53]. However, in other studies, the DM-associated skeletal disorders were not confirmed relative to the non-diabetic population [54–56]. Impaired GH/IGF-1 axis [27, 35] and decreased bone turnover during the period of skeletal growth in adolescents with type 1 diabetes may attenuate formation during this period of bone growth [46–48]. Some other studies conducted in T1DM patients, also showed deficits in bone size, bone strenght and/or lower trabecular and cortical vBMD, compared to healthy controls [57–60]. However, another study found normal levels of bone size [61]. A study in young adults utilising MRI-based measures reported that T1DM patients had deficits in trabecular bone microarchitecture [28], and using the HR-pQCT method found deficits in cortical and trabecular bone [29]. Moreover, these studies identified an association between the presence of microvascular disease and skeletal deficits in patients with T1DM [28, 29]. Uncontrolled T1DM is a known risk factor for bone disease [8–12]. Numerous studies among children and adolescents with T1DM have shown that poorly controlled diabetes may have a negative effect on bone outcomes [49–53].

Body composition significantly influences bone health during diabetic childhood. This study showed that patients with poor glycaemic control (HbA1c > 8%) had a significant impairment of the relationship between the fat and muscle tissue (FM/LBM). Moreover, an increased Z-FM was coupled with decreased lean body mass Z-LBM and Z-TBBMC/LBM ratio (relative bone strength index). It is worth noting that the impairment of the relationship between these components is an important factor in the development of the bone fragility [14, 15] and increased risk of fractures [22–25]. Moreover, other studies showed abnormalities of bone microarchitecture, intra-abdominal fat content, and bone fat marrow [18]. Also regarding the detection of poor glycaemic control, our study observed negative correlations of total body BMD, BMC, and LBM (Z-scores) with increased HbA1c in type 1 diabetes patients. We suggested that the reduced relative bone strength and the high risk of fractures later in life, associated with it, would be higher in these patients. Adolescence is considered a critical period for bone mass accrual. Impaired bone acquisition during this period can lead to alterations in peak bone mass [34, 35, 44, 45], and increase the risk of fractures, but the number of studies corresponding to adolescents is small [25].

In our study, for parameters concerning the lumbar spine, a trend towards decreased BMD and BMC was seen in the group of HbA1c ≥ 8%, but differences were not statistically significant. We have also demonstrated a significant correlation between total body BMC and metabolic control. Similar results were found in a comparison of bone density in T1DM children with the norms of the age- and sex-adjusted general population [51]. However, in this analysis BMD was significantly lower for the lumbar spine as well. The greater difference in BMD and BMC for total skeleton than for lumbar vertebrae between studied groups can evidence an important contribution of decreased mineral density in the peripheral skeleton in T1DM children. This hypothesis should be confirmed in peripheral bone examination by the pQCT method. The use of this imaging technique would allow an assessment of the volumetric BMD, a separate measurement of cortical and trabecular bone, and determination of bone strength.

In this study, in further confirmation of the skeletal and body compartments' assessment, reduced relative bone strength reveals deterioration in the TBBMC/LBM ratio in T1DM patients, suggesting a deterioration of the muscle-bone relationship. BMC is highly influenced by mechanical stimulation from skeletal muscle, consistent with the functional bone-muscle unit theory [43]. The proper muscle-bone interaction during the growth period is an important factor for skeletal adaptation towards changing loads [43]. The decreased BMC and LBM could play an important role in decreased bone strength [31–33]. Our results also demonstrate reduced BMC or BMD Z-scores and an impairment of the relationship between the fat and muscle tissue. Several previous studies also investigated this association and found similar results [16–18, 20, 21, 29, 44–46, 58, 59]. Our observations of reduced relative bone strength of T1DM patients, and more so in the poor metabolic control patients, have led us to speculate that maintaining such status may be associated with increased risk of fractures later in life. Importantly, increased fracture incidence in young patients with T1DM aged <20 years, compared with their control counterparts without T1DM, was observed in a large population-based cohort study [25].

Our study showed that patients with poor glycaemic control (HbA1c > 8%) also had a significant impairment of the relationship between the fat and muscle tissue (FM/LBM). Moreover, an increased fat mass was coupled with decreased lean body mass Z-LBM and Z-TBBMC/LBM (relative bone strength index). It is worth noting that the impairment of the relationship between these components is an important factor in bone fragility [13, 14]. Based on the analysis conducted in the group of patients with good metabolic control, no deficiencies related to bone mineral content or density were found. Also, no impairment in the relationship between the bone and muscle tissue was noted. Interestingly, the value of the TBBMC/LBM parameter (Z-score) in the lumbar spine was higher for patients in this group (HbA1c < 8%). Skeletal development of diabetics during adolescence is also impacted by the metabolic control. Some studies have also shown that poor glycaemic control has negative effects on the circulating IGF system in patients with T1DM [27, 35, 46–48]. Other studies conclude that osteocalcin levels are inversely associated with HbA1c [62, 63], and can be involved in the regulation of glucose and energy metabolism [64]. In this way, the skeleton is a metabolically active organ, and recent studies suggest that there is cross-talk between bone and other tissues [15–17, 22], and osteocalcin plays an important role in the regulation of glucose metabolism [64].

There are many factors that are suggested to impair bone mass accrual in patients with T1DM. In the present study, we found that duration of disease, at a similar age of onset was significantly higher in the poor metabolic group (HbA1c ≥ 8%) compared with the good metabolic group. In the former subgroup, significantly lower Z-TBBMD, Z-TBBMC, and Z-TBBMC/LBM were observed. Although the duration of diabetes was negatively correlated with skeletal outcome, a significant correlation could only be established between longer disease duration and Z-TBBMD, adjusted for confounders. A longer duration of diabetes implies a longer period of elevated glucose levels, and when associated with poor metabolic control, it can have a negative impact on bone mass accrual at an early age [34, 35]. In the pubertal period, fast body weight and height gain takes place [31–33], which in children with T1DM often results in a worsening of metabolic control parameters [5–7]. Indeed, a negative association between disease duration and bone or muscle parameters in adolescents with T1DM has been previously reported [19, 20, 45, 46, 51, 52, 65, 66]. However, other studies have reported normal or minimal deterioration of skeletal outcomes [17, 27, 44, 47, 53, 55, 56, 60, 67]. Of all age groups, adolescents are currently the farthest from achieving HbA1c targets, reflecting the diabetes mismanagement that frequently accompanies the increased independence in diabetes self-care [5].

Several factors could explain the results of our analyses. During the course of type 1 DM, various mechanisms may interact to determine skeletal disorders and impaired bone quality. T1DM is characterised as a state of low bone turnover [68], as determined by osteoblast dysfunction [65–67]. Of note is that insulin deficiency and insulin-like growth factor-1 (IGF-1) reduction [27, 46–48] and hyperglycaemia-induced oxidative stress and accumulation of advanced glycation end-products (AGEs) that compromise collagen properties [69, 70], low osteocalcin levels [62, 63], and/or Wnt signalling pathway [71, 72] seem to be causing these changes, resulting in a reduction in the mature osteoblast numbers and thus bone formation, leading to low peak bone mass at a young age [31–34]. Other factors such as inflammatory systemic diseases are characterised by increased levels of proinflammatory cytokines [73] that uncouple the bone remodelling cycle, also interfering with bone mass acquisition [35]. Consequently, these factors are likely to also have adverse effects on muscle and bone cells and the impaired bone-muscle relationship [20, 21, 40]. Disturbed body composition such as decreased lean muscle mass and increased fat stores [16–18, 38, 39] are a known risk factor of skeletal disorders in DM patients.

To achieve the primary goal of preventing or minimising diabetes complications, it is imperative, among others, to keep blood sugar levels within certain ranges [5]. It is well-recognised that micro- and macrovascular complications are associated with long-term diabetes duration and poor glycaemic control [6, 7]. Close adherence to the dietary balancing of carbohydrate intake and insulin levels [74], healthy eating behaviours as well as a healthy lifestyle are associated with better glycaemic control [75]. Thus, it is necessary to achieve good metabolic control in childhood diabetes in order to ensure appropriate growth and development. The present study aimed at determining the potential benefit of good metabolic control on various skeletal parameters and body composition in T1DM patients. Our results demonstrate that a physiologically relevant well-controlled T1DM in young patients can improve the development of bone mass and that long-term HbA1c < 8% levels are beneficial to bones. Moreover, the effects of vitamin D deficiency on skeletal muscle functioning usually also accompany diabetes, and may be an important factor responsible for decreased muscle mass [76, 77]. As previously reported in the same study population [78], T1DM patients displayed low 25(OH)D serum levels compared to healthy controls (15.3 ± 7.0 vs. 17.9 ± 9.3 ng/mL), but the difference was not significant (*p* > 0.05). Furthermore, higher HbA1c levels coincided with lower 25(OH)D levels. In our previous report, we also showed a close relationship between the altered body composition outcomes and poor glycaemic control in T1DM adolescents. Moreover, we found that increased fat stores (FM Z-score) and reduced lean body mass (LBM Z-score) were inversely associated with 25(OH)D levels in T1DM [78]. Our patients with T1DM showed reduced levels of 25(OH)D compared with the healthy controls. Consistently, in other studies it has been reported that patients with T1DM displayed vitamin D deficiency [11, 20, 28, 47, 54, 59], and that insulin gene expression in pancreatic *β*-cells may also be modulated by vitamin D [20, 35, 37, 76]. Vitamin D also modulates muscle and bone-derived hormones, hormones, including GH-IGF-1 axis and sex hormones, facilitating cross-talk between these tissues [77].

The management of diabetes mellitus in adolescents, in particular with poor metabolic control, is essential. Effective prevention of poor glycaemic control needs comprehensive management [5–7], including healthy lifestyle and adequate nutrition [79, 80], with sufficient vitamin D intake from food and supplements [78]. Some dietary components can impact the mechanisms leading to loss of bone density, skeletal muscle mass, or function, especially if calcium and vitamin D intakes are not adequate. Calcium and vitamin D are well established as essential for bone health as is protein for skeletal muscle, but there are also other nutrients that are integral to bone and muscle physiology and are a key factor in achieving peak bone mass during childhood [31–33]. Thus, optimisation of bone health is critical in paediatric patients, particular for children and adolescents with T1DM. Recommended is an adequate intensive treatment with continuous subcutaneous insulin infusion, balanced healthy diet and self-monitoring of blood glucose, and increasing physical activity. A primary goal of care in adolescent patients with T1DM is the optimisation of glycaemic control for the prevention of bone complications, especially in case of longer disease duration.

Our study has several strengths and weaknesses. The study's strengths are the novelty of studying the skeletal status, body composition, and relative bone strength in the paediatric type 1 diabetes and the comparison of results obtained using the DXA method with a nationally representative group of healthy adolescents, which increases the generalisability of the study findings. The main limitation of this study is its cross-sectional design with respect to establishing temporality in assessing a causal relationship, and other factors such as relatively small sample size, and reliance on a single clinic for the conduct of the work, so the associations of diverse variables should be considered with caution.

Future multicentre, long-term studies are required to verify our results. Future studies should examine this further by increasing the number of patients with T1DM and ensuring a longer study period of glycaemic control. Many factors that link metabolic control and skeletal and body composition have been investigated in the past. A very interesting and still not fully discovered factor impacting bone health as well as type 1 diabetes is the gut microbiome. In the course of diabetes, alterations in the gut microbiome may trigger inflammation of the intestinal mucosa and reduce absorption of essential nutrients. The functional changes of the gastrointestinal tract and gut microbiome in relation to diabetes complications [81] and the interplay between the gut microbiome with the immune system are a highly interesting field of research. Remission prolongation, alleviation of disease, and improving the quality of a patient's life have been highlighted as important objectives in the last years. These could be addressed through a new, promising, therapeutic approach of islet cell transplantation, which has a beneficial impact of restoring partial *β*-cell function [82]. In line with this finding, the new therapy has shown to have a positive impact on diabetes complications. Further development of this innovative approach could benefit patients with T1DM.

## 5. Conclusion

In conclusion, our results showed that adolescent patients with T1DM had lower physiological values of TBBMD, TBBMC, and S24BMD and worse relative bone strength index (TBBMC/LBM ratio), coinciding with higher FM and FM/LBM ratio values compared to age- and sex-adjusted generally healthy counterparts. These observations were further reinforced by decreased LBM values in patients with poor glycaemic control. In contrast, most DXA-assessment parameters appeared to be normal in the well-controlled metabolic group when adjusted for healthy control subjects. Adolescent patients with T1DM should be monitored in terms of their skeletal status and body composition, especially in conditions of poor metabolic control and longer duration of disease. Early recognition of developing abnormalities in skeletal status and body composition is important for bone status in young adulthood when peak bone mass is achieved.

## Figures and Tables

**Table 1:** Demographic and clinical characteristics of patients with T1DM.

Variable^/1^	Total *n* = 60	HbA1c < 8% *n* = 34	HbA1c ≥ 8% *n* = 26	*p* ^∗^
*Clinical, metabolic and anthropometric characteristics*				
Females (%)	32 (53.3)	17 (50.0)	15 (57.7)	NS
Age (y)	15.1 ± 1.9	14.4 ± 1.8	16.0 ± 1.7	0.001
Age at diagnosis (y)	9.9 ± 3.9	10.5 ± 3.3	9.4 ± 4.6	NS
Diabetes duration (y)	5.1 ± 3.9	3.9 ± 3.1	6.7 ± 4.3	0.005
Insulin dose (U/kg/d)	0.75 ± 0.21	0.67 ± 0.16	0.86 ± 0.22	0.001
Pubertal (Tanner stage)	3.7 ± 0.9	3.4 ± 0.9	4.0 ± 0.8	NS
HbA_1c_ (%), 1-year period	7.9 ± 1.4	6.9 ± 0.6	9.2 ± 1.0	0.001
Serum 25(OH)D (ng/mL)	15.3 ± 7.0	17.2 ± 7.9	12.8 ± 4.7	0,015
Serum iPTH (pg/mL)	30.4 ± 16.4	27.0 ± 12.6	34.8 ± 19.8	NS
Serum Ca (mmol/L)	2.44 ± 0.09	2.45 ± 0.10	2.44 ± 0.08	NS
Serum P (mmol/L)	1.39 ± 0.19	1.41 ± 0.21	1.35 ± 0.20	NS
Body height (cm)	166.5 ± 11.5	167.2 ± 12.4	166.2 ± 10.3	NS
Body height (SD score)^/2^	0.07 ± 1.12	0.38 ± 1.10	−0.34 ± 1.02	0.012
Body weight (kg)	58.9 ± 11.9	58.1 ± 13.6	60.0 ± 9.4	NS
Body weight (SD score)^/2^	0.29 ± 1.16	0.39 ± 1.32	0.16 ± 0.93	NS
Physical exercise (hours in school/week)	3.3 ± 1.3	3.3 ± 1.1	3.4 ± 1.5	NS
*DXA parameters*				
TBBMD (g/cm^2^)	1.064 ± 0.093	1.055 ± 0.104	1.074 ± 0.077	NS
TBBMC (g)	2271 ± 516	2252 ± 581	2297 ± 429	NS
LBM (kg)	42.8 ± 10.3	42.8 ± 11.0	42.7 ± 9.7	NS
FM (kg)	13.7 ± 7.4	13.0 ± 8.0	14.6 ± 6.7	NS
S24BMD (g/cm^2^)	1.039 ± 0.166	1.005 ± 0.170	1.084 ± 0.154	NS
S24BMC (g)	43.3 ± 11.9	42.1 ± 12.5	44.8 ± 11.2	NS
S24BMC/LBM (ratio)	1.019 ± 0.197	0.984 ± 0.185	1.063 ± 0.207	NS
TBBMC/LBM (ratio)	0.054 ± 0.007	0.053 ± 0.006	0.055 ± 0.008	NS
FM/LBM (ratio)	0.343 ± 0.198	0.324 ± 0.202	0.370 ± 0.194	NS

^/1^Data are expressed as mean ± SD; ^/2^SD-scores calculated according to age- and sex-dependent reference data for healthy subjects; ^∗^*p*-values for differences between subgroups were calculated with independent Student's *t*-test, or Chi-square test for categorical variables; NS — not significant. TBBMD — total body bone mineral density; TBBMC — total body bone mineral content; LBM — lean body mass FM — fat mass; S24BMD — bone mineral density, lumbar spine L2–L4; S24BMC — bone mineral content, lumbar spine L2–L4; S24BMC/LBM — bone mineral content, lumbar spine L2–L4/lean body mass ratio; TBBMC/LBM — total body bone mineral content/lean body mass ratio; FM/LBM — fat mass/lean body mass ratio.

**Table 2:** The Z-score values for DXA parameters in T1DM patients with well or poorly controlled glycaemia.

Variable (Z-scores)^/1^	*x* ± SD	SE	95% CI	*p* ^∗^
*n* = 60				
Z TBBMD	−0.38 ± 0.95	0.12	−0.63; −0.14	0.003
Z TBBMC	−0.29 ± 0.97	0.13	−0.54; −0.03	0.027
Z LBM	−0.08 ± 0.90	0.12	−0.32; +0.15	0.483
Z S24BMD	−0.30 ± 1.06	0.14	−0.58; −0.03	0.031
Z S24BMC	−0.09 ± 0.99	0.13	−0.35; +0.16	0.461
Z TBBMC/LBM	−0.29 ± 0.97	0.13	−0.54; −0.04	0.025
Z S24BMC/LBM	+0.29 ± 1.16	0.15	−0.01; +0.59	0.055
Z FM^/2^	+0.38 ± 1.27	0.16	+0.05; +0.70	0.025
Z FM/LBM	+0.31 ± 0.99	0.13	+0.63; +0.57	0.018
*n* = 34 (*HbA1c* < 8%)				
Z TBBMD	−0.10 ± 0.97	0.17	−0.44; +0.23	0.535
Z TBBMC	+0.01 ± 1.02	0.18	−0.35; +0.37	0.959
Z LBM	+0.20 ± 0.90	0.15	−0.11; +0.52	0.197
Z S24BMD	−0.20 ± 1.16	0.20	−0.61; +0.20	0.321
Z S24BMC	+0.09 ± 1.06	0.18	−0.28; +0.46	0.618
Z TBBMC/LBM	−0.07 ± 0.96	0.17	−0.41; +0.27	0.677
Z S24BMC/LBM	+0.44 ± 1.20	0.21	+0.02; +0.86	0.039
Z FM^/2^	+0.36 ± 1.44	0.25	−0.14; +0.86	0.153
Z FM/LBM	+0.20 ± 1.05	0.18	−0.16; +0.57	0.266
*n* = 26 (*HbA1c* ≥ 8%)				
Z TBBMD	−0.74 ± 0,80	0.16	−1.07; −0.42	0.001
Z TBBMC	−0.67 ± 0.77	0.15	−0.98; −0.37	0.001
Z LBM	−0.45 ± 0.77	0.15	−0.77; −0.14	0,006
Z S24BMD	−0.43 ± 0.91	0.18	−0.80; −0.07	0.022
Z S24BMC	−0.34 ± 0.85	0.17	−0.68; +0.01	0.053
Z TBBMC/LBM	−0.58 ± 0.92	0.18	−0.95; −0.20	0.004
Z S24BMC/LBM	+0.10 ± 1.10	0.22	−0.35; +0.54	0.652
Z FM^/2^	+0.40 ± 1.03	0.20	−0.02; +0.81	0.060
Z FM/LBM	+0.45 ± 0.90	0.18	+0.09; +0.82	0.018

*x* ± SD: mean ± standard deviation; SE: standard error of the mean; 95% CI: confidence interval; ^∗^*p*-values were calculated with one-sample *t*-test compared to the hypothetical values of 0 (expected in healthy subjects); ^/1^Z-scores for DXA parameters calculated according to age- and sex-dependent reference data for healthy subjects; ^/2^Wilcoxon test TBBMD — total body bone mineral density; TBBMC — total body bone mineral content; LBM — lean body mass; FM — fat mass; S24BMD — bone mineral density, lumbar spine L2–L4; S24BMC — bone mineral content, lumbar spine L2–L4; S24BMC/LBM — bone mineral content, lumbar spine L2–L4/lean body mass ratio; TBBMC/LBM — total body bone mineral content/lean body mass ratio; FM/LBM — fat mass/lean body mass ratio.

**Table 3:** Relationship between disease duration, insulin requirement, glycaemic control, and skeletal and body composition Z-score values (for age- and sex- dependent reference data for healthy subjects) in T1DM patients.

Variable (Z-scores)^/1^	Diabetes duration		Insulin requirement		HbA1c	
	*r*	*p*	*r*	*p*	*r*	*p*
Z TBBMD	−0.260^∗^	0.049	−0.423^∗^	0.001	−0.283^∗^	0.031
Z TBBMC	−0.253	0.056	−0.302^∗^	0.021	−0.331^∗^	0.011
Z S24BMD	−0.163	0.221	−0.324^∗^	0.013	−0.133	0,321
Z S24BMC	−0.121	0.356	−0.242	0.067	−0.246	0.063
Z TBBMC/LBM	−0.158	0.238	−0.386^∗^	0.003	−0.182	0.171
Z S24BMC/LBM	−0.094	0.481	−0.272^∗^	0.039	−0.129	0.334
Z LBM	−0.246	0.062	−0.110	0.410	−0.345^∗^	0.008
Z FM	0.046	0.731	−0.111	0.408	0.013	0.921
Z FM/LBM	0.123	0.359	−0.115	0.390	0.054	0.689
Diabetes duration (y)	—	—	0.303^∗^	0.021	0.265^∗^	0.045
Insulin requirement (U/kg/d)	0.303^∗^	0.021	—	—	0.626^∗^	0.001
HbA1c (%)	0.265^∗^	0.045	0.626	0.001^∗^	—	—

Results are expressed as correlation coefficient (*r*) and two-tailed statistical significance (*p*); *p*-values adjusted for age, sex, pubertal stage, height, weight, and physical activity level; ^∗^*p*-value ≤ 0.05; ^/1^Z-scores for DXA parameters calculated according to age- and sex-dependent reference data for healthy subjects; TBBMD — total body bone mineral density; TBBMC — total body bone mineral content; LBM — lean body mass; FM — fat mass; S24BMD — bone mineral density, lumbar spine L2–L4; S24BMC — bone mineral content, lumbar spine L2–L4; S24BMC/LBM — bone mineral content, lumbar spine L2–L4/lean body mass ratio; TBBMC/LBM — total body bone mineral content/lean body mass ratio; FM/LBM — fat mass/lean body mass ratio.

## Data Availability

The data analysed during this study are included in the published article. The numerical data used to support the findings of this study are available from the corresponding author upon reasonable request.

## Abbreviations

TBBMD: Total body bone mineral density
TBBMC: Total body bone mineral content
LBM: Lean body mass
FM: Fat mass
S24BMD: Bone mineral density, lumbar spine L2–L4
S24BMC: Bone mineral content, lumbar spine L2–L4
S24BMC/LBM: Bone mineral content, lumbar spine L2–L4/lean body mass ratio
TBBMC/LBM: Total body bone mineral content/lean body mass ratio
FM/LBM: Fat mass/lean body mass ratio
HbA1c: Glycated haemoglobin A1c.
